# A pilot study on metabolomic characterization of human glioblastomas and patient plasma

**DOI:** 10.21203/rs.3.rs-2662020/v1

**Published:** 2023-03-10

**Authors:** Allison Liu, Orwa Aboud, Lina A. Dahabiyeh, Orin Bloch, Oliver Fiehn

**Affiliations:** University of California, Davis; University of California, Davis; The University of Jordan; University of California, Davis; University of California, Davis

**Keywords:** glioblastoma, untargeted metabolomics, biomarker, feasibility, pilot

## Abstract

**Purpose:**

To determine whether recurrent GBMs are metabolically distinct from primary GBM, and whether patient plasma can be used as a liquid biopsy to reflect this difference.

**Methods:**

In a single center cohort study, tissue and blood samples from 15 patients with glioblastoma (9 glioblastoma tissues at diagnosis, 3 pairs of tissue, and 6 pairs of plasma specimens at diagnosis and at recurrence) were analyzed.

**Results:**

Several metabolites had significant alternations in both tumor and plasma specimens. In the tissue, the following representative metabolites had a significant increase in peak intensity at recurrence compared to diagnosis: N-alpha-methylhistamine (p = 0.037), glycerol-3-phosphate (p = 0.029), phosphocholine (p = 0.045), and succinic acid (p = 0.025). In patient plasma, metabolites that significantly increased at recurrence included: 2,4-difluorotoluene (p = 0.031), diatrizoic acid (p = 0.032), indole-3-acetate with (p = 0.029), urea (P = 0.025), pseudouridine (p = 0.042), and maltose (p = 0.035). Metabolites that significantly decreased in plasma at recurrence were: eicosenoic acid (p = 0.017), glucose-1-phosphate (p = 0.017), FA 18:2 (linoleic acid) (p = 0.017), arginine (p = 0.036), fatty acids 20:3 (homo-gamma-linolenic acid (p = 0.036), galactosamine (p = 0.007), and FA 18:3 (linolenic acid) (P = 0.012). Principal component analysis showed that the metabolomic profiles differ between tumor tissue and patient plasma.

**Conclusions:**

Our data suggest that metabolomic profiles of human GBM tissue and patient plasma differ at diagnosis and at recurrence. Many metabolites involved in tumorigenesis and metabolomic flexibility were identified. A larger study using targeted metabolomic assay is warranted to measure the levels of these metabolites, which will help identify the metabolomic signatures in both GBM tissue and patient plasma for risk stratification, clinical outcome prediction, and development of new adjuvant metabolomic-targeting therapy.

## Introduction

Glioblastoma (GBM) is a fatal tumor with a median survival of less than 2 years.[1] Most GBMs respond to initial therapeutic interventions of surgical resection and chemoradiation[2, 3] but eventually, all patients will relapse. The range of progression free survival is wide, from a few months to more than 2 years. Understanding the differences in molecular features of GBM and its microenvironment at diagnosis and at recurrence can help identify biomarkers to monitor for treatment response, understand pathogenesis of treatment resistance, and predict outcome. It may also help identifying new therapeutic targets.

Surgical resection and standard chemoradiation improve survival but this initial effect is limited by the development of resistance.[4] While therapy resistance and the aggressiveness of GBM have been investigated at the genomic and transcriptomic levels, less is known about the metabolic phenotypes. Altered metabolism is a hallmark of cancer.[5] Metabolomic changes are critical for tumor cells to undergo conversion to aggressive and treatment-resistant phenotypes.[6] Recurrent, therapy resistant tumors develop within the high dose radiation field, and the ability of recurrent tumors to resist therapy is, in part, due to metabolomic alternation within the tumor[7]. Tumor metabolism is influenced by both cancer cell-intrinsic information (genome, epigenome, proteome, post-translational modifications) and cell-extrinsic cues from the tumor microenvironment.

Targeting the metabolome has succeeded in a number of cancers including high-grade gliomas.[7] Glucose uptake can inform prognosis in a variety of cancers including glioma.[8] Metabolomic profiling in GBM tissue may provide important information on the differences in tumor responses to initial standard of care therapies and for understanding the differences between tumors at diagnosis and at recurrence. This may help predict tumor aggressiveness and patient prognosis. Early data from several metabolomic studies in small cohorts of GBM patients have yielded promising results.[9, 10] GBM is a heterotrophic tumor but is also known to have highly heterogeneous lipid metabolism[11] and favors heterotrophy[12, 13]. However, the comprehensive metabolomic profile including metabolites, biogenic amines, and lipidomes have not been studied. The comparison between GBM tissue and patient blood specimens has not been performed. Therefore, it is unclear if patient blood can be used as a surrogate to predict tumor status in the brain.

We performed a study to test the hypothesis that recurrent GBMs are metabolically distinct from GBM at initial diagnosis, and patient plasma can be used as a liquid biopsy to reflect this difference. Using untargeted mass spectrometry, we profiled the metabolomes, lipidomes, and biogenic amines of human glioblastoma tissue and patient plasma both at diagnosis and at recurrence, and correlated metabolomic information with clinical data. We identified patterns of metabolomic remodeling in tumor tissue and patient plasma. These changes can pave the way for metabolomic signature identification for treatment response monitoring, risk stratification, and outcome prediction.

## Methods

### Glioblastoma tissue specimens

The UC Davis Pathology Biorepository at the Comprehensive Cancer Center provides high quality and well-characterized human brain tumor tissue specimens. In this centralized biorepository, all samples were collected after patients’ informed consents and underwent quality control by a clinical neuropathologist. From January 2010 to July 2022, a total of 12 fresh frozen GBM specimens were identified and obtained from the biorepository for metabolomic analysis. Limited deidentified clinical information was abstracted from the medical records within the scope of the approved IRB protocol of the biorepository.

### Plasma collection

The UC Davis Department of Neurosurgery (Dr. Orin Bloch Laboratory) has an IRB approved protocol to collect blood samples from GBM patients at diagnosis and throughout treatment, along with access to clinical information of these patients. All procedures performed in this study were in accordance with the 1964 Helsinki Declaration and its later amendments or comparable ethical standards. From January 2018 to July 2022, a total of 12 plasma specimens were selected for metabolomic analysis.

### Untargeted metabolomic, biogenic amine, and lipidomic analyses for GBM tissue and patient plasma

#### Sample preparation and extraction

Metabolites and biogenic amines were extracted as previously described.[14] Blood plasma or serum was extracted following the protocols first published by V. Matyash et al.[15] Using this protocol, lipid extracts in methyl tert-butyl ether phase (MTBA) were separated from proteins and polar hydrophilic small molecules (in the methanol/water phase) in a way that the lipids were found in the top layer of liquid-liquid separations, rather than in the bottom layer. Decanting the top layer therefore ensured that the extracts were not contaminated by proteins or polar compounds. The top layer was used for lipidomics while the bottom layer (methanol/water phase) was very suitable for the hydrophilic interaction liquid chromatography-mass spectrometry (HILIC-MS) investigations.

### Data Acquisition

Metabolite profiling using HILIC-MS was performed on the Agilent 1290 UHPLC/Sciex TripleTOF 6600 mass spectrometer. Metabolites (5 μL) were separated using Waters AcquityUPLC BEH amide column (1.7μm, 2.1 × 50 mm) and a binary mobile phase (solvent A: 100% LC-MS grade H2O with10 mM ammonium formate and 0.125% formic acid; solvent B 95:5 (*v/v*) ACN:H2O with 10 mM ammonium formate with 0.125% formic acid). Data were acquired in data-dependent acquisition mode with a mass range 50–1500 *m/z* for MS1 and 40–1000 *m/z* for MS2.

Lipidomic data were acquired using the Agilent 1290 UHPLC/Agilent 6530 QTOF (for positive mode) and 6550 QTOF (for negative mode) mass spectrometer. Waters Acquity Premier BEH C18 column (1.7μm, 2.1 × 50 mm) was used for chromatographic separation applying binary mobile phase system (Positive mode: mobile phase A: 60:40 v/v acetonitrile:water + 10 mM ammonium formate + 0.1% formic acid; mobile phase B: 90:10 v/v isopropanol:acetonitrile + 10 mM ammonium formate + 0.1% formic acid. Negative mode: mobile phase A: 60:40 v/v acetonitrile:water + 10 mM ammonium acetate; mobile phase B: 90:10 v/v isopropanol:acetonitrile + 10 mM ammonium acetate). MS scan range and mass resolution for positive mode were 120–1200 *m/z* and 10,000, respectively, and 60–1200 *m/z* and 20,000 for negative mode.

Primary metabolism data were acquired by gas chromatography (GC)-MS using an Agilent 7890A GC coupled to a Leco Pegasus HT TOF mass spectrometer as previously described. [14] Briefly, extracts were dried down, derivatized by methoxyamination and trimethylsilylation, and injected in splitless mode with a temperature gradient from 50–330°C. Mass spectra were acquired at 17 Hz from 85–500 Da.[14]

### Raw data processing and metabolite annotation

Acquired raw LC–MS and LC-MS//MS data were processed as previously described.[14] Raw CSH-C18-TOF (for lipidomics) and HILIC-TTOF (for polar metabolites profiling) MS data were processed using MS-Dial 4.9, data-independent MS/MS deconvolution for comprehensive metabolome analysis, for untargeted peak-picking, peak alignment and annotation of related peaks.[14] Raw GC-TOF MS data files were processed using ChromaTOF and metabolomics BinBase database.[14]

### Statistical analysis

Descriptive statistics were used to characterize baseline patient and treatment characteristics. Individual metabolite abundance comparisons at diagnosis and at relapse were performed using GraphPad Prism 9 (version 9.5, San Diego, CA). MetaboAnalyst 5.0 MetaboAnalyst 5.0 (McGill University, Montreal, QC, Canada) (http://www.metaboanalyst.ca) [16] was used to generate principal component analysis (PCA), score plots, heat maps and volcano plots. The processed peak heights with their annotation were imported to MetaboAnalyst, normalized to the total sample median and auto scaled. Paired or unpaired Student’s *t*-Test was used to identify significantly altered metabolites between the compared groups (*p*-value of less than 0.05 was considered significant). ChemRICH (Chemrich.fiehnlab.ucdavis.edu), a statistical enrichment approach based on chemical similarity rather than sparse biochemical knowledge annotations was used to group the metabolites. ChemRICH sets have a self-contained size where p-values do not rely on the size of a background database.

## Results

### Cohort description

Patient demographics are described in Table 1. In the tissue cohort, a total of 12 specimens from 9 patients were analyzed (9 specimens at diagnosis, 3 of which had paired specimens at recurrence). The mean age at diagnosis was 49 years. There was a male predominance of 67%. Most of this cohort was non-Hispanic White in race/ethnicity.

In the plasma cohort, a total of 12 paired specimens (diagnosis and at recurrence) from 6 GBM patients were analyzed. The mean age was 54 years. There was a male predominance of 67%. The original GBM tissue pathology all showed *IDH* wild type. Additional pathology features including *EGFR*, *MGMT*, and *ATRX* status are shown in Table 1. The mean progression free survival of this cohort was 14 months. The mean overall survival was 17 months.

### Unsupervised exploratory analysis on GBM tissue metabolomic, biogenic amine, and lipidomic profiling

**Figure 1:**
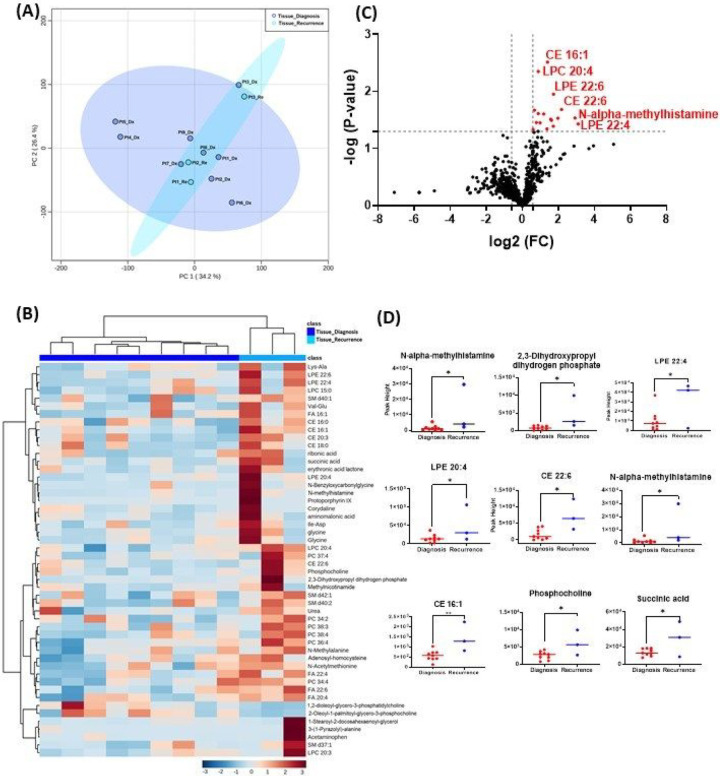
Comparison of metabolomic profiles of 9 glioblastoma tumor tissue specimens at diagnosis and 3 tissue specimens at recurrence. (A) Principal component analysis (PCA) plot showing different trends of metabolomic characteristics between tissues at diagnosis (purple) and tissue at recurrence (light blue). (B) Heatmap of the top 50 altered metabolites at diagnosis and at recurrence. Blue indicates decreased peak value and maroon indicates increased peak value of each compound listed. (C) Volcano plot of up regulated metabolites in red and down regulated metabolites in blue in glioblastoma tumor tissue specimens at recurrence comparing to at diagnosis using p-value of <0.05 and fold change cutoffs of 1.5. (D) Plots of individual values for each metabolite demonstrating peak value changes at diagnosis and at recurrence in brain tumor tissue. Values were determined as peak heights from LC/MS analysis. Single asterisk indicates a p value of <0.05. Double asterisks indicate a p value of <0.01.

The GBM tissue cohort included 9 tumor tissue specimens at diagnosis, 3 of which had paired tissue specimens at recurrence. A complete list of significantly altered metabolites is available in Supplemental Table 1. A summary of these changes is shown in Fig. 1. Principal component analysis (PCA) showed two grouped clustering trends with significant overlap (Fig. 1, A). A heat map of the top 50 significantly altered metabolites, lipids, and biogenic amines, showed differences in cluster trends between tissue samples at diagnosis and at recurrence (Fig. 1, B). There were several significantly upregulated compounds (Fig. 1, C) in the lipidomic and biogenic amine analysis. Also included in Fig. 1 are scattered column plots for compounds with significant change in abundance at diagnosis and at recurrence (Fig. 1, D), which included N-alpha-methylhistamine (P = 0.037), 2,3-dihydroxypropyl dihydrogen phosphate with (P = 0.029), CE22:6 (P = 0.021), LPE 20:4 (P = 0.004), LPE 22:6 (P = 0.011), LPE 22:4 (P = 0.041), CE 20:3 (P = 0.031), CE 16:1 (P = 0.003), phosphocholine with a (P = 0.045), and succinic acid (P = 0.025).

**Figure 2:**
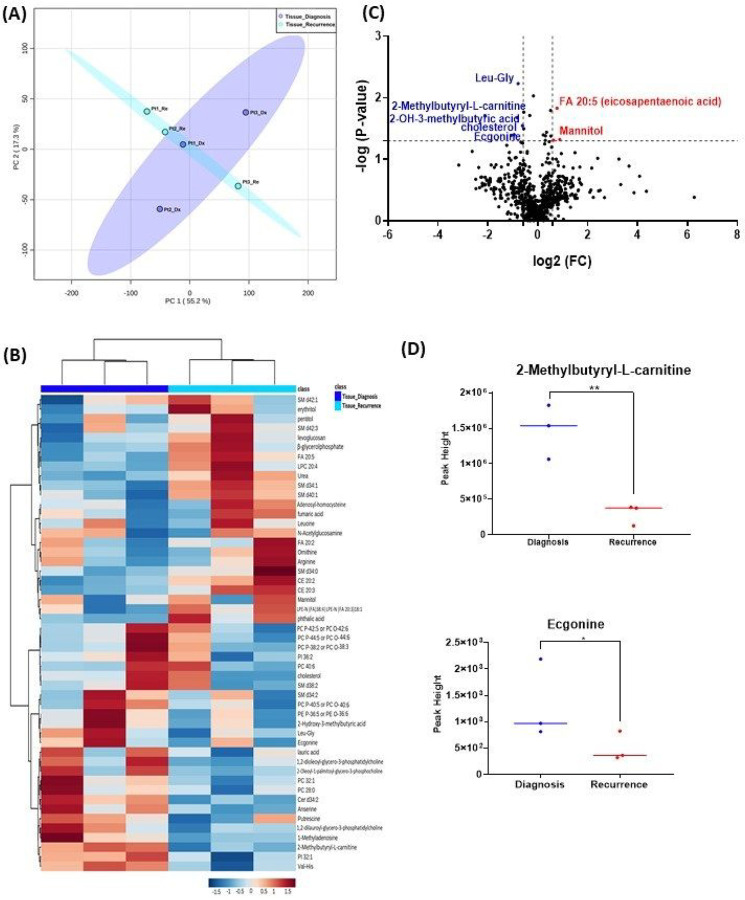
Comparison of metabolomic profiles of 3-paired fresh frozen brain tumor tissue specimens at diagnosis and at recurrence. (A) Principal component analysis (PCA) plot showing different trends of metabolomic characteristics between tissues at diagnosis (purple) at tissue at recurrence (light blue). (B) Heatmap of the top 50 altered metabolites. Blue indicates decreased peak value and red indicates increased peak value of each compound listed. (C) Volcano plot of up regulated metabolites in red and down regulated metabolites in blue in glioblastoma tumor tissue specimens at recurrence comparing to at diagnosis using p-value of <0.05 and fold change cutoffs of 1.5. (D) Plots of individual values for each metabolite demonstrating peak value changes at diagnosis and at recurrence in brain tumor tissue. Values were determined as peak heights from LC/MS analysis. Single asterisk indicates a p value of <0.05. Double asterisks indicate a p value of <0.01.

When analyzing the three paired tissue specimens at diagnosis and at recurrence, again the PCA plot demonstrated two overlapping clusters (Fig. 2, A). The heat map showed more visible separation between tissue samples at diagnosis and at relapse (Fig. 2, B). We found 19 compounds that were significantly altered. Volcano plot (using p-value < 0.05 and Fold Change cutoff 1.5) revealed that 3 metabolites were upregulated, and 6 metabolites were down regulated (Fig. 2, C). A complete list of significantly altered metabolites is available in Supplemental Table 2. Among these metabolites, we found that the level the 2-methylbutyryl-L-carnitine (P = 0.02) and an unknown compound with eluting at 1.84 minutes with accurate mass 186.1075 Da (P = 0.04) were significantly higher in primary tumors than recurrent GBM (Fig. 2, D).

### Unsupervised exploratory analysis on plasma metabolomic, biogenic amine, and lipidomic characteristics

**Figure 3:**
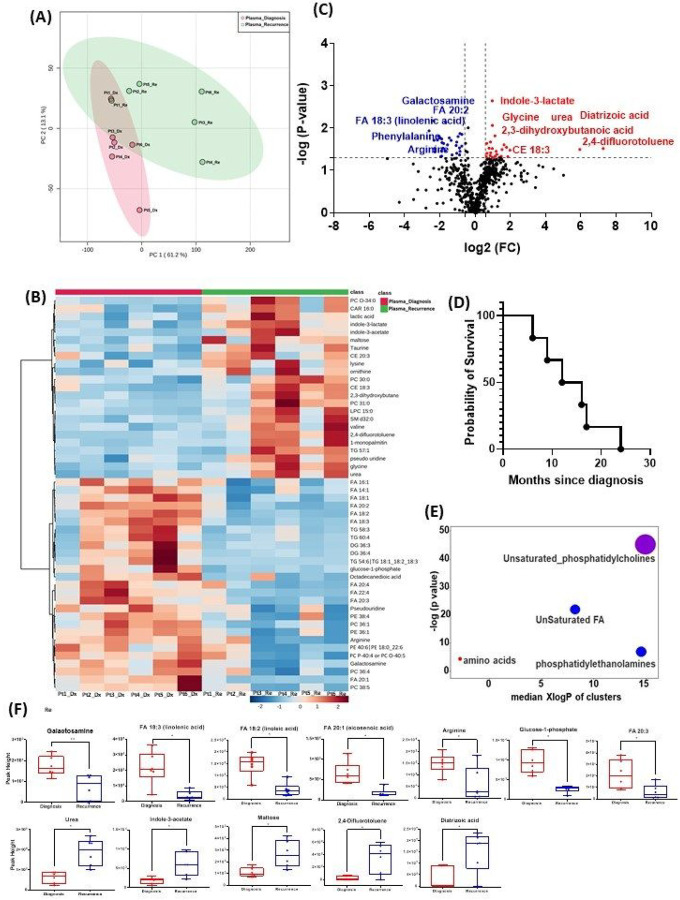
Comparison of metabolomic profiles in plasma of glioblastoma patients at diagnosis and at recurrence. (A) Principal component analysis (PCA) plot showing different trends of metabolomic characteristics between tissues at diagnosis (purple) at tissue at recurrence (light blue). (B) Heatmap of the top 50 altered metabolites. Blue indicates decreased peak value and red indicates increased peak value of each compound listed. (C) Volcano plot of up regulated metabolites in red and down regulated metabolites in blue in glioblastoma tumor tissue specimens at recurrence comparing to at diagnosis using p-value of <0.05 and fold change cutoffs of 1.5. Single asterisk indicates a p value of <0.05. Double asterisks indicate a p value of <0.01. (D) Progression free survival of 6 patients whose plasma samples were analyzed. (E) Significantly altered metabolite clusters by ChemRich, a statistical enrichment approach based on chemical similarity. The size of the dots is in proportion with the level of alteration in each cluster of metabolites. Red indicates increased peak value and blue indicates decreased peak value. (F) Box plots demonstrating metabolite peak value changes at diagnosis and at recurrence in brain tumor tissues. Values were determined as peak height from LC/MC analysis. Single asterisk indicates a p value of <0.05. Double asterisks indicate a p value of <0.01.

All 6 patients enrolled in this cohort had paired plasma specimens at diagnosis and at recurrence. However, these were not the same patients whose GBM tissue were studied in the cohort above. PCA analysis showed evident separation between the metabolomic profiles at diagnosis and at recurrence, except for patient 1, whose metabolomic profiles were similar at diagnosis and at recurrence (Fig. 3, A). The heatmap of the top 50 altered metabolites showed visible differences between plasma at diagnosis and at recurrence (Fig. 3, B). Again, the metabolite profile of patient #1 at recurrence was similar to its status at diagnosis, while the other patients’ profiles demonstrated differences.

**Figure 4:**
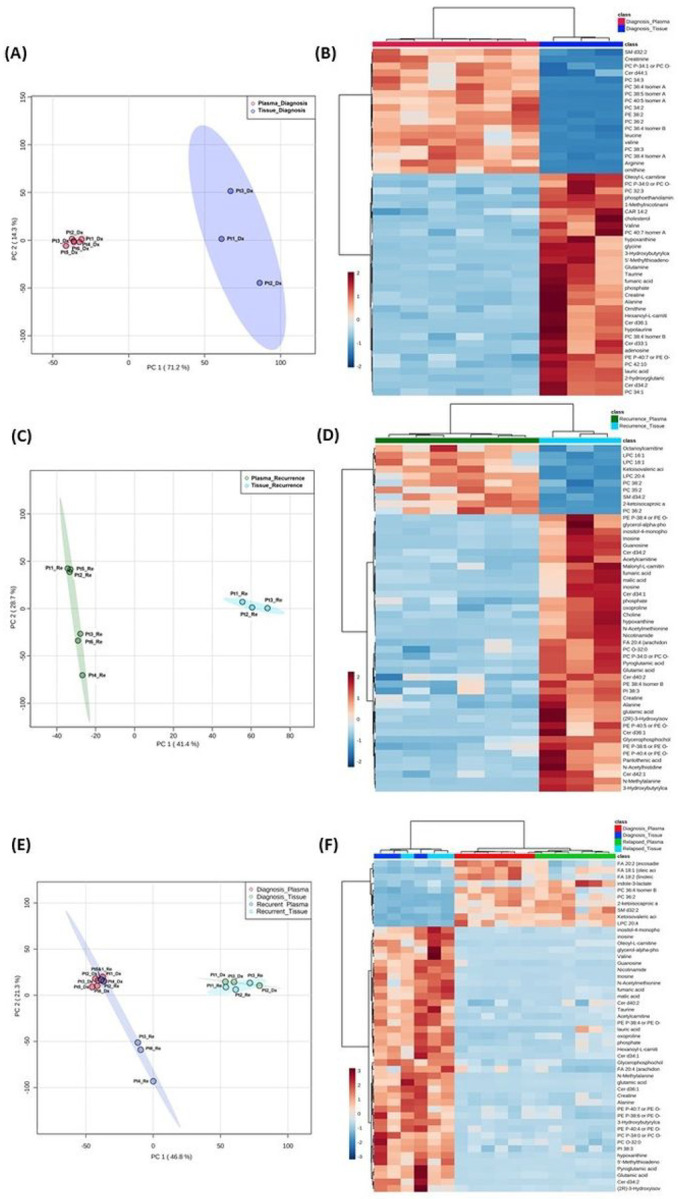
Comparison of metabolomic profiles in glioblastoma tumor tissue and patient plasma. (A, C, E) Principal component analysis plots comparing tissue vs plasma specimens at diagnosis, tissue vs plasma at recurrence, and all four specimen groups. (B, D, F) Heatmaps of the top 50 significantly altered metabolites in the comparisons correlating with A, C, and E, respectively.

There were 61 compounds that were altered significantly between diagnosis and recurrence in the patient plasma. The representatives were shown in Fig. 3, C. A complete list of significantly altered metabolites is available in Supplemental Table 3. The progression free survival of these 6 patients was shown in Fig. 3, D. Using ChemRich, we were able to identify that based on chemical structural similarity, amino acids and unsaturated phosphatidylcholines were significantly up-regulated and unsaturated fatty acids and phosphatidylethanolamines were down-regulated (Fig. 4, E). The compounds with significantly increased abundance at recurrence included 2,4-difluorotoluene (P = 0.031), diatrizoic acid (P = 0.032), indole-3-acetate with (P = 0.029), urea (P = 0.025), pseudo uridine (P = 0.042), and maltose (P = 0.035). The compounds with significantly decreased abundance included FA 20:1 (eicosenoic acid) (P = 0.017), glucose-1-phosphate (P = 0.017), FA 18:2 (linoleic acid) (P = 0.017), arginine (P = 0.036), FA 20:3 (homo-gamma-linolenic acid) (P = 0.036), galactosamine (P = 0.007), and FA 18:3 (linolenic acid) (P = 0.012) (Fig. 3, F).

### Combined analyses on glioblastoma tissue and patient plasma at diagnosis and at recurrence

Both the PCA and heatmap analyses showed separate metabolic profiles from tissues at diagnosis and plasma at diagnosis (Fig. 4, A and B). Similarly, the metabolomic profile from tissue at recurrence separated from plasma at recurrence (Fig. 4, C and D). When placing all four groups of data in one plot (Fig. 4, C and D), we again saw inter-specimen differences between the metabolomic profiles in tissue and plasma, but there were no intra-specimen differences at diagnosis and at recurrence.

## Discussion

In this study, we investigated the comprehensive untargeted metabolomic, lipidomic, and biogenic amine profiles of GBM tissue and patient plasma specimens at diagnosis and at recurrence. Despite a small overall cohort size, our result showed that many metabolites were altered in GBM tissue and patient plasma at recurrence when compared to diagnosis. Our study demonstrated the feasibility of studying GBM tissue and patient blood specimen longitudinally using metabolomic methodology.

GBM display marked metabolic heterogeneity in their microenvironments.[17] Both glucose and lipid metabolisms are abnormally regulated in GBM tissues.[18, 19] In our study, we observed several metabolites that had changed in abundance at recurrence when compared to diagnosis. Many of these metabolites were also identified in a recently published study on the metabolic hallmarks of gliomas.[20] Specifically, we identified 2-methylbutyryl-L-carnitine and ecgonine that were known to reflect tumor metabolic flexibility in brain tumor tissues at diagnosis and at recurrence. Carnitine serves as a “shuttle-molecule” that allows fatty acid acyl moieties to enter the mitochondrial matrix for oxidization via the beta-oxidation pathway[21]. We found that the 2-methylbutyryl-L-carnitine level was significantly reduced in recurrent tumors compared to initial GBM tissue. Carnitine transporter modulation has been thought to be a potential target for cancer treatment.[21] In addition, we also found many altered levels of lipids in GBM tissue at recurrence when compared to initial diagnosis. Our findings are in line with previously published data suggesting lipid metabolic alterations in GBM.[22] In addition, mannitol was upregulated in recurrent tissue compared to the original tumor, suggesting BBB permeability changes after surgical resection and chemoradiation. This may suggest mannitol as a vehicle to guide targeted treatment.

The list of metabolites with significantly altered abundance and fold changes differ when comparing the unpaired samples (9 tissue samples at diagnosis vs 3 tissue samples at recurrence) and the paired samples (3 tissue samples from the same patients at diagnosis and at recurrence). Also, despite a small sample size, the paired tissues samples at diagnosis and at recurrence demonstrate a clearer trend in the differences in compound abundance. Therefore, paired samples are recommended in future studies given their better capacity as an internal control.

In both the PCA plot from patient plasma and the heatmap generated from the top 50 altered metabolites, we found that the metabolomic profiles differed between specimens at diagnosis and at recurrence, except for one patient with early recurrence. The metabolomic profile of this patient’s plasma at diagnosis was different from the rest of the plasma specimens at diagnosis and was similar to the group pattern at recurrence. This interesting finding needs to be validated in a larger cohort of patients with treatment refractory tumors and early recurrence. This metabolomic signature may indicate a high risk and poor prognosis.

In the plasma cohort, we found several significantly altered metabolites with large fold changes. For example, 2,4-difluorotoluene increased in patient plasma at recurrence; this metabolite is incorporated into DNA and undergoes replication by DNA polymerase enzymes.[23] The change suggests rapid growth of recurrent tumors. Diatrizoic acid also increased at recurrence. However, this is a contrast agent used during imaging. Indole-3-acetate is an indol-3-yl carboxylic acid anion and has a role as a human metabolite. We again found several other compounds involved in glucose and lipid metabolism. The overall pattern changes of these compounds need to be validated in targeted metabolomics for their potential candidacy as biomarkers for treatment response and tumor recurrence. The enrichment of these metabolites in recurrent GBM tissue may suggest that targeting metabolic activity can be a potential adjuvant targeted treatment for GBM patients.

Interestingly, when plotting all four groups in the same PCA plot, the brain tissue and plasma specimens separated distinctly from each other regardless of disease status (Fig. 4). There was no overlap between specimen types and, the intra-specimen comparison at diagnosis and at recurrence became smaller. This suggested that plasma metabolite patterns are not reflective of brain tumor tissue, and therefore, two sets of biomarker panels are necessary for tissue and plasma. The pattern of tumor microenvironment can be further altered through end organ metabolism in the plasma. Also, plasma is affected by systemically administered medication for peri-operative care.

Our study has limitations. First, we must acknowledge that the sample size in this pilot study is small. Second, although we have paired data within the brain tissue and plasma cohorts at diagnosis and at recurrence, we were unable to identify if the tissue and plasma specimens were from the same patients due to the restrictions of the UC Davis biorepository consents for GBM tissue. In addition, we did not have normal brain tissue or plasma for comparison. These limitations prevented us from identifying small changes in metabolite abundance. Therefore, only significant and large fold changes were analyzed. A bigger cohort with paired specimens will be emphasized in future studies. The current study paved the way for the next targeted metabolomics, lipidomic, and biogenic amine studies validating and further investigating these profiles.

## Conclusions

Our data suggest that metabolomic profiles of human GBM tissue and patient plasma differ at diagnosis and at recurrence. Many metabolites involved in tumorigenesis and metabolomic flexibility were identified. A larger study using target metabolomic assay is warranted to measure the levels of these metabolites, which will help identify the metabolomic signatures in both GBM tissue and patient plasma for risk stratification, clinical outcome prediction, and development of new adjuvant metabolomic-targeting therapy.

## Figures and Tables

**Table 1:** Patient characteristics

Total cohort n = 15		
GBM tissue, n = 9		
Age at diagnosis (years, range)	49 (31–60)	
Sex	Male	6 (67%)
	Female	3 (33%)
Race/Ethnicity	Non-Hispanic White	6 (67%)
	Hispanic/Latino	2 (22%)
	Not reported	1 (11 %)
Plasma, n = 6		
Age at diagnosis (years)	54 (43–60)	
Sex	Male	4 (67%)
	Female	2 (33%)
Race/Ethnicity	Non-Hispanic White	4 (67%)
	Hispanic/Latino	1 (17%)
	Black	1 (17%)
IDH	Wild type	6 (100%)
	Mutant	0
EGFR	Amplification	3 (50%)
	Negative	3 (50%)
MGMT	Methylated	3 (50%)
	Unmethylated	3 (50%)
ATRX	Retained	6 (100%)
PFS (months, range)	14 (6–24)	
OS (months, range)	17 (9–25)	

## Data Availability

The datasets generated during and/or analyzed during the current study are available from the corresponding author on reasonable request.
